# Weak Cation Selectivity in HCN Channels Results From K^+^-Mediated Release of Na^+^ From Selectivity Filter Binding Sites

**DOI:** 10.1093/function/zqac019

**Published:** 2022-04-22

**Authors:** Daniel Bauer, Jan Wissmann, Anna Moroni, Gerhard Thiel, Kay Hamacher

**Affiliations:** Department of Biology and Centre for Synthetic Biology, TU Darmstadt, Schnittspahnstrasse 3, 64287 Darmstadt, Germany; Department of Physics, TU Darmstadt, Schlossgartenstrasse 7, 64289 Darmstadt, Germany; Department of Biosciences, University of Milan, via Celoria 26, 20133 Milan, Italy; Department of Biology and Centre for Synthetic Biology, TU Darmstadt, Schnittspahnstrasse 3, 64287 Darmstadt, Germany; Department of Biology and Centre for Synthetic Biology, TU Darmstadt, Schnittspahnstrasse 3, 64287 Darmstadt, Germany; Department of Physics, TU Darmstadt, Schlossgartenstrasse 7, 64289 Darmstadt, Germany

**Keywords:** HCN channels, ion selectivity, molecular dynamics, free energy, rate theory

## Abstract

Hyperpolarization-activated cyclic nucleotide-gated (HCN) channels generate the pacemaker current which plays an important role in the timing of various biological processes like the heart beat. We used umbrella sampling to explore the potential of mean force for the conduction of potassium and sodium through the open HCN4 pore. Our data explain distinct functional features like low unitary conductance and weak selectivity as a result of high energetic barriers inside the selectivity filter of this channel. They exceed the 3-5 kJ/mol threshold which is presumed as maximal barrier for diffusion-limited conductance. Furthermore, simulations provide a thermodynamic explanation for the weak cation selectivity of HCN channels that contain only two ion binding sites in the selectivity filter (SF). We find that sodium ions bind more strongly to the SF than potassium and are easier released by binding of potassium than of another sodium. Hence ion transport and selectivity in HCN channels is not determined by the same mechanism as in potassium-selective channels; it rather relies on sodium as a weak blocker that can only be released by potassium.

## Graphical Abstract

**Graphical Abstract:**
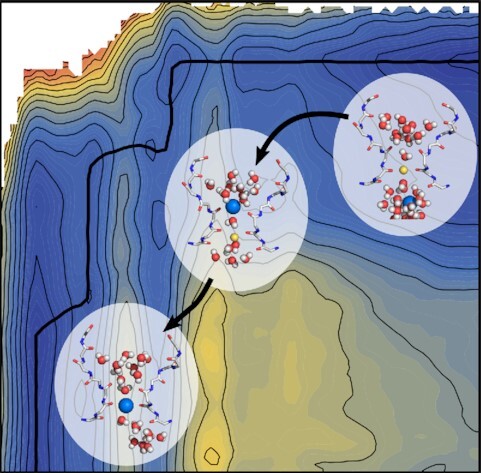

## Introduction

Hyperpolarization-activated cyclic nucleotide-gated (HCN) channels are encoded by the closely related HCN1-4 gene family. They are expressed in cells of the heart and the brain^1–3^ where they generate the pacemaker current, also known as funny current. The latter plays a crucial role for the timing of various biological processes including heart beat (I_f_ current) and rhythmic firing of neurons (I_h_ current).^4,5^ Another important role of HCN channels is their ability to reduce the refractory period of neurons in the cerebellum by attenuating strong hyperpolarization. This in turn reduces the resting time between subsequent action potentials.^6^

HCN channels have a fourfold symmetry with the canonical 6TM/P (6 transmembrane helices, 1 pore) architecture of voltage-gated potassium channels. TM5-TM6 form in each subunit the pore domain with the pore-Helix and SF. TM5-TM6 are located N-terminally and form the voltage-sensing domain (VSD) connected via a short linker to the pore domain (PD). In HCN, the VSD is packed against the PD of the same subunit in a so called “non-swapped” arrangement.^7,8^ HCN channels are hyperpolarization-activated; downward movement and tilting of TM4 of the VSDs at negative voltages open the central pore domain.^8–10^ Finally, the open probability of HCN channels is further modulated in an allosteric manner by binding of cyclic adenosine monophosphate (cAMP). Ligand binding to the C-terminal cyclic-nucleotide binding domain (CNBD) shifts the channels half-activation voltage V_1/2_ to less negative values.^11–13^ The cytosolic N-terminal HCN domain contributes to transmit the signal of cAMP-binding to the VSD.^14^

**Figure 1.**
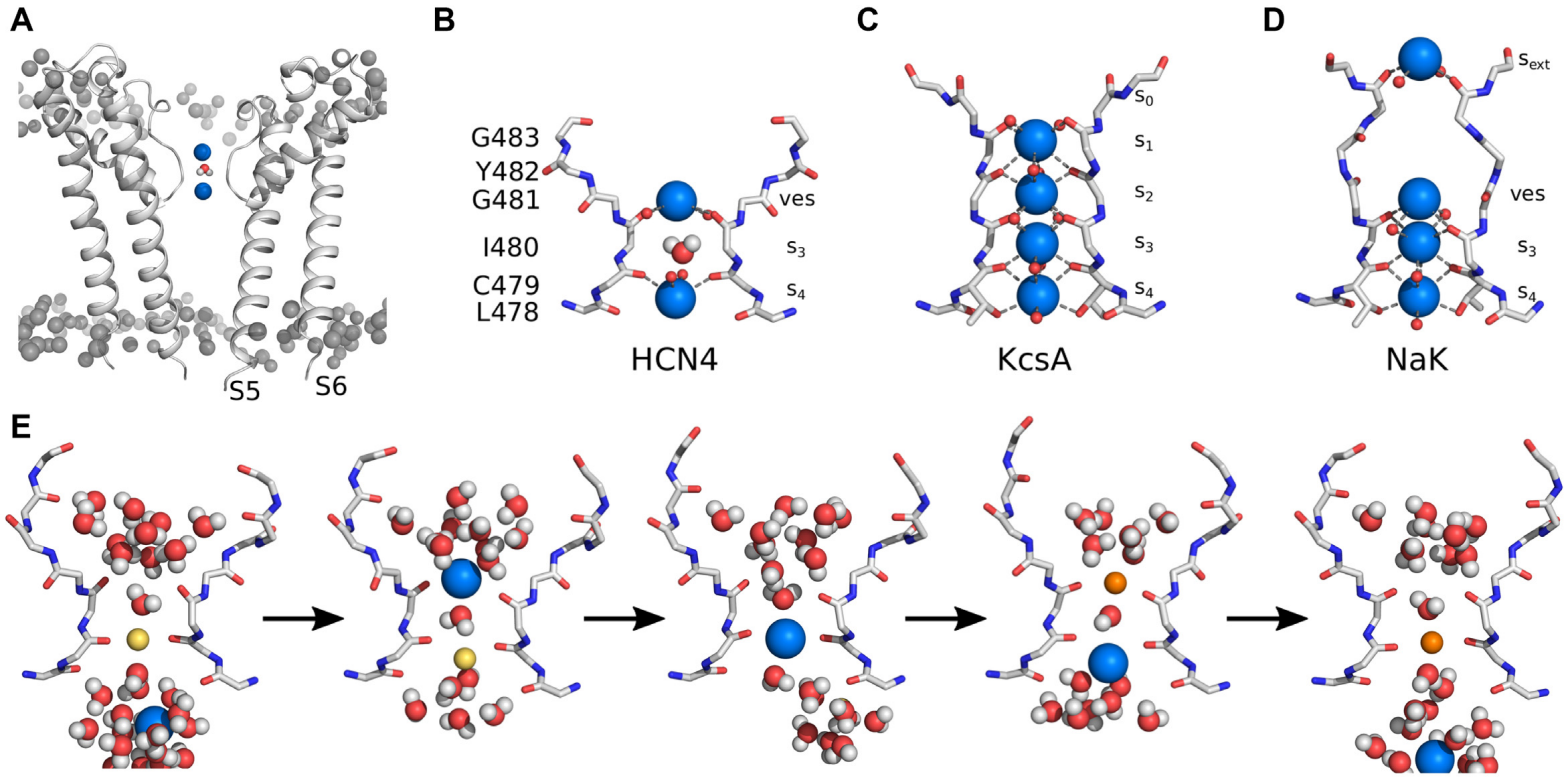
**(A)** Side view of the two opposing subunits of the PD of HCN4 embedded in a lipid bilayer with labeled transmembrane helices S5-S6. Grey spheres represent lipid headgroups. Potassium ions and water inside the SF are shown as blue and red/white spheres, respectively. **(B)** Close-up view of two subunits of the HCN4 SF with labeled amino acids (L478 to G483), binding sites (s_3_, s_4_), and extracellular vestibule (ves). **(C)** The SF of the K^+^-selective KcsA (PDB: 1K4C) with binding sites s_0_ to s_4_ and (**D**) the non-selective NaK (PDB: 3E8H) with binding sites s_ext_, s_3_, s_4_ and the water filled vestibule (ves). Interactions between the SF backbone and potassium ions are shown as dashed lines. Oxygen atoms of hidden subunits are shown as small red spheres. **(E)** Mixed ion conduction in HCN4. A single Na^+^ ion is initially bound to carbonyl oxygens of C479. Binding of K^+^ to carbonyl oxygens of I480 displaces the Na^+^ into s_4_. Subsequently, K^+^ replaces Na^+^ at the lower binding site and the initial one-ion configuration is reset.

Even though the SF of HCN channels contains the GYG signature sequence, which is common to all selective potassium channels, HCN channels discriminate only moderately between K^+^ and Na^+^ with a preference of 2–6:1^1–3^,^15,16^ This weak K^+^/Na^+^ selectivity guarantees an influx of Na^+^ into the cells upon HCN activation, which in turn generates a progressive depolarization that drives the free running voltage to the threshold for a subsequent action potential. A comparison of the canonical SF motif of the selective KcsA channel with the respective domain of HCN4 revealed by Cryo-EM (Figure 1a, b) gives a possible explanation for the low cation selectivity of these channels. The SF of K^+^-selective channels like KcsA (sequence: TVGYG, Figure 1c) adopts a conformation with 4 binding sites (s_1_ to s_4_) in which one K^+^ ion is coordinated by 8 oxygens from the protein backbone carbonyls.^17^ These interactions effectively mimic the hydration shell of an ion while moving through the SF. A fifth binding site (s_0_), where ions are only partially desolvated, has been identified above s_1_ in the entrance to the SF18. In contrast, the cyo-EM structures of HCN1 and HCN4 (sequence: CIGYG, Figure 1b) show that their SFs are indistinguishable^8^ independent on the fact that one is closed and isolated in the presence of KCl^7^ while the other is open and was isolated in NaCl.^8^ Both SFs adopt a conformation in which only two of the canonical binding sites (s_3_ and s_4_) are provided by carbonyl oxygens of L478, C479 and I480.^7,8^ Carbonyl oxygens of the G481, which would form s_2_ in a canonical K^+^ channel, are rotated away from the central axis. Binding sites s_0_ to s_2_ are thus replaced by a funnel-shaped, fully-solvated vestibule (ves) at the extracellular side of the SF. Additionally, since a Thr in the conserved filter sequence of selective K^+^ channels is in HCN channels replaced by Cys (C479 in HCN4), the s_4_ site is in the latter channels significantly wider than the corresponding site in highly K^+^-selective channels; while this side measures 8.9 Å in HCN4, it is only 5.3 Å in KcsA. This structural peculiarity of HCN channels presumably further reduces selectivity in these proteins and leaves their SF with only a single canonical binding site s_3_. With these structural features, the SF of HCN resembles more closely the SF of the non-selective NaK channel (Figure 1d). Also, this channel shows a similar enlargement above the two K^+^-binding sites (s_3_ and s_4_). But different from HCN channels, the NaK channel contains an additional ion binding site (s_ext_) upstream of the vestibule and the s_4_ site is shaped similar to the corresponding site in K^+^ channels.^19^ Interestingly, even though NaK has more ion binding sites than HCN channels, it is still less K^+^-selective than HCN channels. The reason for this is presumably related to the ability of the NaK channel to adopt different conformations that facilitate either K^+^ or Na^+^ conductance.^20,21^ Molecular dynamics simulations performed on the experimentally obtained open-state pore of HCN4^8^ and on the computationally opened pore of HCN1^22^ revealed a unique SF conformation with ion binding and conduction mechanisms different from both K^+^ selective channels and the non-selective NaK channel. In HCN4 and HCN1, K^+^ prefers binding to the carbonyl planes of residues C479 and I480 (Figure 1e)—similar to what has been reported for Na^+^ binding in canonical K^+^ channels.^23,24^ The resulting conduction mechanism is an alternation of two distinct states: a single ion, either K^+^ or Na^+^, occupies the C479 carbonyl plane and is coordinated by 4 carbonyl oxygens and two water molecules residing in the canonical binding sites s_3_ and s_4_ (Figure 1e). When a new ion arrives and binds to the carbonyls of I480, the lower ion is displaced into s_4_. In this configuration, both ions are only partially desolvated as they are in close contact with water from the central cavity or SF vestibule, respectively. Once the lower ion leaves the SF, the upper ion moves through s3 and resets the initial state by binding to the C479 plane.^8^ It is striking that over several µs of simulation and multiple conduction events, no notable occupation of s_3_ was observable for either ion species, even though the dimensions of s_3_ closely resemble those observed in selective K^+^ channels.

To better understand the permeation and selectivity mechanisms of HCN channels, we calculated the potential of mean force (PMF) associated with the conduction of K^+^ and Na^+^ inside the SF of HCN4. This provides information on the conduction mechanism from an energetic perspective and gives insight into how the SF architecture of HCN favors different selectivity and conduction kinetics than the equivalent structure of highly selective K^+^ channels.

## Methods

### MD Simulations and Equilibration

Previous MD simulations with the pore domain recapitulated the main functional features of HCN4 channels determined from experiments.^8^ This included the low unitary conductance, weak K^+^/Na^+^ selectivity and absence of channel conductance in pure NaCl. Based on these data and the general assumption that ion selectivity in K^+^ channels is determined only by the pore domain,^25^ we decided to follow the same strategy here: molecular dynamics simulations were performed using the PD (residues 412–523) of HCN4 (PDB: 7NP3) embedded into a pre-equilibrated and solvated 1-palmitoyl-2-oleoyl-sn-glycero-3-phosphocholine (POPC) bilayer. Initial simulation systems were built with the CHARMM-GUI webserver.^26,27^ Then, two ions, either K^+^ or Na^+^, separated by a single water molecule were placed in the selectivity filter or central cavity. The negative charge on the protein (8e^–^) was then balanced by replacing 6 randomly selected solvent molecules with 6 additional K^+^. Final systems consisted of the protein, 14 778 water molecules, 182 lipids, 6 K^+^ and any combination of 2 additional K^+^ or Na^+^ inside the SF. To obtain sufficiently equilibrated starting structures for free energy calculations, systems were energy minimized (steepest descend, 2000 steps) and equilibrated (100 ps NVT, 20 ns position-restrained NPT). Restraints were then gradually lifted (3 ns) followed by further equilibration for 100 ns. During equilibration, the 2 ions inside the SF were prevented from leaving it via repulsive potential walls (k = 10,000 kJ/mol/nm^2^) and further ions were prevented from entering the pore by the same mechanism.

All simulations were carried out with GROMACS 2019^28,29^ in combination with Plumed 2^30^ and the AMBER99sb*-ILDN force field.^31,32^ The TIP3P water model,^33^ Berger-derived POPC lipids^34^ and improved ion parameters by Joung and Cheatham^35^ were used. Van-der-Waals interactions were cut-off at 1 nm and electrostatics were treated by PME^36^ beyond 1 nm. Temperature and pressure were kept at 310 K and 1 bar using the stochastic V-rescale Thermostat^37^ and Parrinello-Rahman Barostat,^38^ respectively. All bonds were restraint using LINCS^39^ and hydrogen atoms were represented as virtual sites to allow for an integration time step of 4 fs.^40^ Like Kopec and coworkers^41^ we also applied a restrain on a set of distances on the bottom half of the S6 helix to prevent any unwanted closing of the cytosolic gate after isolating the experimentally determined open pore from its regulating domains. This operation is justified considering that any conformational transition of the protein at the gate–if they at all happen–is much slower than the short simulation times, which are required for the ion transitions.

### Umbrella Simulations

Two-dimensional potential of mean forces (PMFs) were calculated using self-learning adaptive umbrella-sampling (US) simulations^42^ starting from configurations differing in their initial ion placement: K^+^/K^+^ (2x), K^+^/K^+^/K^+^ (2x), Na^+^/Na^+^/Na^+^ (2x), Na^+^/K^+^/K^+^ (2x) or K^+^/Na^+^/Na^+^ (2x) (Figure S1, Table S1). A harmonic biasing potential with a force constant of k = 1000 kJ/mol/nm^2^ was applied to the z-coordinate of ions relative to the center-of-mass (COM) of oxygens of C478 and I480 (z = 0 nm). For the two ions system (K^+^/K^+^), US boundaries were set to −0.35 to 1.85 nm and −1.55 to 0.55 nm for the position of the upper ion (z_1_) and lower ion (z_2_), respectively (Figure S2). For the three ion systems (K^+^/K^+^/K^+^, Na^+^/Na^+^/Na^+^, Na^+^/K^+^/K^+^ and K^+^/Na^+^/Na^+^), different US boundaries were used with a maximal range of −0.35 to 2.45 nm and −2.25 to −0.35 nm for the position of the upper ion (z_1_) and the geometric center of the two lower ions (z_2,3_), respectively. The US interval was 0.1 nm for all systems. Simulations were run for at least 5 ns using the NVT ensemble and the first 0.5 ns of each window was omitted from analysis. Additional longer simulations were run at regions with low sampling efficiency and along the minimal free energy pathway (MFEP) when required. The results of two individual US runs with matching ion species were unbiased together using the weighted histogram analysis method with 100 bins in both dimensions and a convergence tolerance of 10^–6^ kJ/mol.^43,44^ The total number of individual simulations used for unbiasing where 444 (K^+^/K^+^), 715 (K^+^/K^+^/K^+^), 633 (Na^+^/Na^+^/Na^+^), 436 (Na^+^/K^+^/K^+^) and 619 (K^+^/Na^+^/Na^+^). Possible one-dimensional free energy paths were extracted from interpolated 2D PMFs via the nudged elastic band (NEB) method^45^: therefore, MFEPs were calculated between all minima of the 2D PMF using NEB. These short snippets were then joined together to construct possible paths connecting start and end points of the transition pathway. Resulting paths were further minimized and ranked according to their energy. Representative snapshots of individual configurations along the pathways were extracted from US simulations by clustering trajectories based on the coordinates of ions.

### Rate Calculation

1D projections of the MFEP were used to calculate the relative total transition rates of K^+^ and Na^+^ through the HCN4 channel. From the projected MFEP, transition rates between neighboring intermediate configurations were calculated using Kramers method for over-damped systems.^46,47^ We calculated transition rates r_i, j_ between two neighboring minima i and j via

$$\begin{equation*}
{\rm{\ }}{r_{i,j}} = \frac{{\omega \left( {{\lambda _i}} \right)\omega \left( {{\lambda _j}} \right)}}{{2\pi \gamma }}\ {e^{\frac{{ - \Delta F_{i,j}^\ddagger }}{{{k_B}T}}}}
\end{equation*}
$$

where ω(λ_i_) is the curvature of the MFEP around the minima λ_i_, γ is the friction coefficient and ΔF^‡^_i, j_ is the height of the energy barrier. We approximated ω(λ_i_) by fitting a cubic function on each peak and valley.^48^ Since our systems are similar, we assumed that the friction γ is the same for every system. Therefore, by considering only relative transition rates between systems, γ cancels out.

The total transition rate r_1, n_ over n minima was then derived by chaining singular reaction rates similar to chemical reaction rate equations.^49^ The system starts from a pure single state concentration and traces the propagation through the other configurations. Here, we assumed that only the MFEP is relevant for the conduction process and other, less likely, paths were excluded. Also, only transitions between adjacent configurations are allowed and the final state is absorbing to simplify the equations:

$$\begin{equation*}
{\lambda _1} \mathbin{\lower.3ex\hbox{$\buildrel\textstyle\rightarrow\over {\smash{\leftarrow}\vphantom{_{\vbox to.5ex{\vss}}}}$}} {\lambda _2} \mathbin{\lower.3ex\hbox{$\buildrel\textstyle\rightarrow\over {\smash{\leftarrow}\vphantom{_{\vbox to.5ex{\vss}}}}$}} ... \mathbin{\lower.3ex\hbox{$\buildrel\textstyle\rightarrow\over {\smash{\leftarrow}\vphantom{_{\vbox to.5ex{\vss}}}}$}} {\lambda _{n - 1}} \to {\lambda _n}
\end{equation*}
$$

This allowed us to derive a set of ordinary differential equations (ODEs).

$$\begin{equation*}
\begin{array}{@{}*{1}{c}@{}} {\frac{d}{{dt}}\ {p_1} = {r_{2,1}}\ {p_2} - {r_{1,2}}{p_1}}\\ {\frac{d}{{dt}}\ {p_2} = {r_{1,2}}\ {p_1} + {r_{3,2}}{p_3} - \left( {{r_{2,1}} + {r_{2,3}}} \right){p_2}}\\ {...}\\ {\frac{d}{{dt}}\ {p_n} = {r_{n - 1,n}}\ {p_{n - 1}}} \end{array}
\end{equation*}
$$

where p_i_ is the concentration at configuration i. The ODEs were solved numerically using SciPy.^50^ The concentration for the final configuration was tracked over time and converted in to the total transition rate by calculating the expected value for r_1, n_.

## Results and Discussion

A common strategy to investigate the dynamics underlying ion channel conductance in proteinaceous pores is the sampling of a multidimensional PMF. This reveals possible low- and high energy states associated with a set of initially chosen collective variables (CVs) and allows to extract a MFEP as most probable route through the free energy surface. To study ion conduction, a natural choice for CVs is the position of individual ions along the principal axis (z) of the channel. The movements of these ions (here either K^+^ or Na^+^) represent the most important degrees of freedom in the otherwise rather static system of the SF. In the open pore of the HCN4 channel, the SF alternates between two major states with one or two bound ions and one additional ion in the central cavity.^8^ With these CVs (labeled z_1_ to z_3_ from here on), the PMF basically follows the trajectory of the HCN mediated inward current from the extracellular medium into the central cavity below the SF. In our analysis, we first consider a system with only two ions inside of the pore (K^+^/K^+^ system) and compare it to a system with an additional ion inside the central cavity (K^+^/K^+^/K^+^ system) to investigate the influence of the cavity ion. This scenario with only K^+^ ions is then compared to the case with only Na^+^ ions (Na^+^/Na^+^/Na^+^ system). Finally, the analysis is extended to systems with mixed ion species (Na^+^/K^+^/K^+^ and K^+^/Na^+^/Na^+^ system).

### High Energy Barriers During K^+^ Conduction

**Figure 2.**
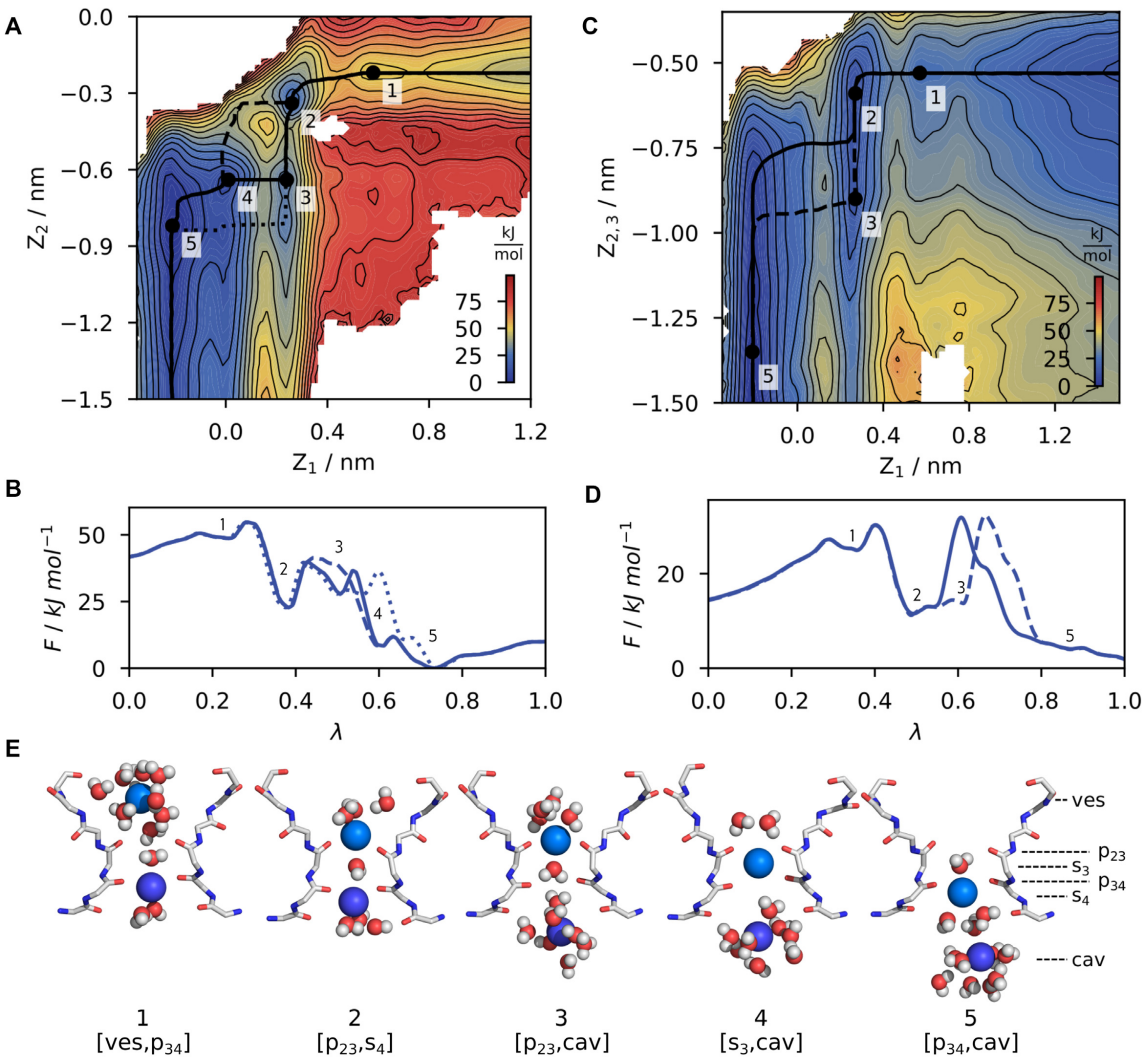
Free energy landscape of potassium ion conduction through the HCN4 pore. **(A)** Free energy landscape in the K^+^/K^+^ system. The Free energy is shown as function of the position of two ions z_1_ and z_2_ along the principal axis of the channel. The s_3_ binding site is located at z = 0 nm and more positive values of z correspond to locations at the extracellular side. Black solid and dashed lines represent possible paths through the 2D landscape. Local minima of the MFEP are labeled numerically (1–5) and are cross-referenced in subplots b&e. Contours represent a free energy difference of 5 kJ/mol. **(B)** 1D projection of the MFEP from (A) with labeled minima 1–5 along the reaction coordinate lambda following inward conduction. **(C-D)** Similar to (A-B), but for the K^+^/K^+^/K^+^ system. **(E)** Snapshots of the SF corresponding to the free energy minima 1–5.

Figure 2A and B show the 2D PMF and 1D projections of free energy paths following ion conduction in a system where z_1_ and z_2_ represent the positions of two K^+^ ions in the filter (K^+^/K^+^ system). Configurations representing local minima of the PMF are cross-referenced in Figure 2E and labeled according to the positions of the ions. For example [ves, p_34_] corresponds to a configuration of the SF with the upper ion in the vestibule and the lower ion bound to p_34_. In general, MFEPs for inward transition of K^+^ are in good agreement with MD simulations published earlier for the same channel.^8^ During a transition from the vestibule (ves), through the SF into the central cavity (cav), ions visit carbonyl oxygen planes p_23_ and p_34_ as well as canonical binding sites s_3_ and s_4_. The conduction mechanism thereby follows a sequence of states labeled 1–5 that connect the two major minima with either two ions (state 2, [p_23_, s_4_]) or a single ion (state 5, [p_34_, cav]) inside the pore (Figure 2E). With a single ion bound to p_34_ (state 1, [ves, p_34_]), a newly arriving ion can fit into the vestibule above the SF where it keeps its first hydration shell. When the newly arriving ion then binds to p_23_, it displaces the lower ion from the p_34_ plane towards s_4_ (state 2, [p_23_, s_4_]). In this configuration, both ions are still partially solvated and can interact with water molecules from the vestibule and the central cavity, respectively. Following the MFEP (solid line), the lower ion then leaves s_4_ into the central cavity (state 3, [p_23_, cav]). Finally, the upper ion moves through the shallow minima in s_3_ (state 4, [s_3_, cav]) to p_34_ (state 5, [p_34_, cav]), resetting the initial state with a single ion bound to the SF motif. Interesting to note is that the PMF seemingly allows the system to explore several alternative pathways in addition to the MFEP: starting from state 2, [p_23_, s_4_] ions can also move in concert between [p_23_, s_4_] and [p_34_, cav] (dashed line between state 2&4, Figure 2A). Also, the upper ion can hold its position while the ion in the cavity already starts leaving the pore (dotted line between state 3&5, Figure 2A). These paths show comparable energy barriers (Figure 2B) and should thus also be observable in classical MD trajectories. With this prediction, we revisited our already published simulation data on HCN4 conductance^8^ and where indeed able to find in a few cases and only at moderate voltages (−250 mV) individual transition events in which ions are not moving in the dominant concerted manner but with a small delay (Figure S3). This confirms that in principle slightly different mechanisms are indeed possible for K^+^ conduction in HCN4. The exact conduction mechanism therefore appears to be mainly driven by a high bias of ions to move to the p_34_ plane. This state can be reached via various alternative pathways through the energy landscape.

Comparison of the free energy surfaces from calculations with two and three ions (Figure 2a and Figure 2c, respectively) reveals that ion conduction follows in both systems the overall same mechanism albeit with significant differences in free energies. The actual energy barriers between states 1&2 and states 2&5 are of similar height for both systems with a difference < 2 kJ/mol (Figure S5), which we assume to be close to the accuracy of our calculations. However, conductance comes with a much higher overall gain in free energy in the K^+^/K^+^ system compared to K^+^/K^+^/K^+^. This is especially evident when comparing the free energy difference between start and end states of the MFEPs. Here, the free energy difference ΔF_1→5_ for transition between state 1&5 is very high (−51 kJ/mol) in the K^+^/K^+^ system and lower (−25 kJ/mol) in the K^+^/K^+^/K^+^ systems (Figures S4 and S5). Once the state 5 is reached, the systems must “invest” the same amount of energy for continuous conduction e.g., to move from state 5 over the periodic boundary of the system back to state 1. This high energy barrier represents the process in which the lower ion is leaving the central cavity, while a newly arriving ion approaches the SF from the extracellular side. The finding that an ion in the cavity is able to lower this barrier suggests that the conductance of HCN channels is not only determined by transition of the ion through the filter but also by ion occupancy of the cavity.

Transition state theory (TST) is a simple technique that can be used to estimate free energy barriers from experimentally observed rate constants. For HCN4, the lowest estimate for conductance is 6.05×10^5^ s^–1^ (0.97 pS^51^), which corresponds to a single free energy barrier of 40 kJ/mol. This value can be seen as an upper estimate of observable free energy barriers for any transition of the system. Even in systems with multiple free energy barriers, none of them can exceed this estimated limit in the frame of the experimentally measured unitary conductance. Hence, it is apparent that only the K^+^/K^+^/K^+^ system fulfills this requirement with a ΔF^‡^_5→1_ of 30 kJ/mol. Here, we calculated the height of the free energy barrier for a transition over the periodic boundary of the system between state 5 and the maximum before state 1 of the MFEP. In the K^+^/K^+^ system the respective ΔF^‡^_5→1_ value exceeds with 51 kJ/mol this barrier; the latter resembles more a channel with a sub-pS conductance. We interpret these differences between the systems with two or three K^+^ in the following way: structurally, the conduction mechanism of the two systems differs only in the presence of an ion inside the central cavity when the filter switches between the one-ion and two-ion configuration. Therefore, the additional barrier in free energy in the K^+^/K^+^ between states 5 and 1 likely resembles the free energy for inserting this third ion into the central cavity below the SF.

The data underscore that the cavity of HCN channels always contains at least on ion that only leaves it when a new one arrives from the SF. This conclusion is in good agreement with simulation data in which the cavity of HCN4 is constantly found populated by an ion.^8^ It is possible that this is a peculiar feature of inward-rectifying K^+^ channels because a similar ion occupancy of the cavity was observed in simulations of other potassium inward rectifiers.^52,53^ In contrast, outward-rectifying K^+^ channels can exhibit, at least for short time, an ion-free cavity after the cavity ion has entered the SF for K^+^ efflux.^41,54,55^ This highlights the importance of the presence of a cavity ion for subsequent conduction steps in HCN channels. This conclusion is also in agreement with simulation data that predict the constant presence of an ion in the cavity of HCN4.^8^ A similar ion occupation of the cavity was also made in other inwardly-rectifying potassium channels.^52,53^

Since the system with two ions predicts an unrealistically high energy barrier for the transition between states 5 and 1, the following analysis will only refer to systems with three ions in the PD. In previous studies on other K^+^ channels (KcsA, MthK), it has been proposed that energy barriers in the SF need to be < = 3 to 5 kJ/mol in order to support the nearly diffusion-limited conductance in these channels.^18,56^ Scrutiny of the respective PMFs of HCN4 shows that even in the PMF with three ions, relevant states are separated by much higher energy barriers (Figure 2c, d). Both major transitions between states 2 and 5 and states 5 and 1 are separated by free energy barriers of ΔF^‡^ > 20 kJ/mol, values well above the 3 to 5 kJ/mol found in other channels. These exceptionally high energy barriers from free energy simulations provide an explanation for the particularly low unitary conductance of HCN channels, which are known both from single channel recordings as well as computer simulations.^8,51^,^57–59^ Hence the low unitary conductance of HCN channels reflects the need of ions to overcome much higher energy differences for conduction. This kinetically slows down ( = high barriers) ion transitions compared to related K^+^ channels with a high(er) unitary conductance.

### Reduced Conductance With Only Sodium

**Figure 3.**
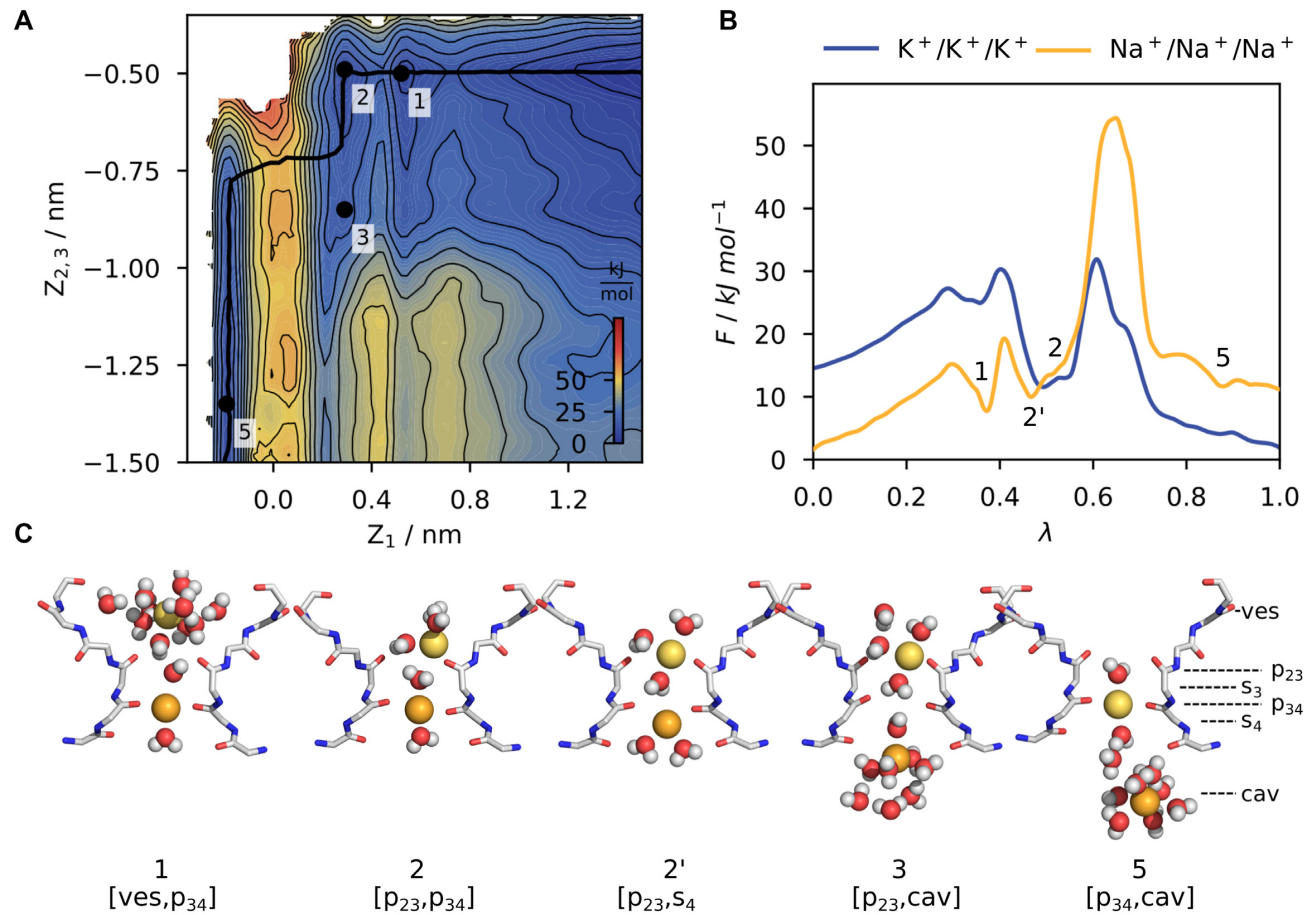
**(A)** Free energy landscape of sodium ion conduction. The free energy is shown as a function of the position of three ions z_1_ and z**2,3** along the principal axis of the channel. The s_3_ binding site is located at z = 0 nm and more positive values of z correspond to locations at the extracellular side. Contours represent a free energy difference of 5 kJ/mol. The black solid line represent the MFEP and local minima are labeled numerically (1–5) and are cross-referenced in subplots b&c. **(B)** Comparision of MFEPs in the Na^+^/Na^+^/Na^+^ (orange) and K^+^/K^+^/K^+^ (blue) system. Lambda is the reaction coordinate along the respective MFEP and minima of the Na^+^/Na^+^/Na^+^ PMF are labeled accordingly. **(C)** Snapshots of the SF corresponding to the free energy minima 1–5.

The MFEP of sodium conduction in the Na^+^/Na^+^/Na^+^ system follows a similar scheme to that observed in systems with potassium (K^+^/K^+^ and K^+^/K^+^/K^+^, Figure 3). The PMF has a minimum of potential energy with a single ion bound to p_34_. A second Na^+^ can then potentially enter the vestibule and bind to p_23_. In contrast to the K^+^/K^+^ system, this does not immediately result in a displacement of the lower Na^+^ into s_4_. Instead, in a pure sodium conduction, state 2’ [p_23_, p_34_] and state 2 [p_23_, s_4_] are of similar energy with no noticeable energy barrier in between. This resembles the general tendency of Na^+^ to bind to carbonyl planes instead of acquiring the square antiprismatic coordination presented by a cage of 8 oxygens. With two ions bound, the lower ion can leave the SF fairly easily, leading to state 3 [p_23_, cav]. However, the MFEP does not follow this route; it rather suggests that ions move almost in concert when the lower ion leaves the SF and the upper ion takes its position at p_34_, leading to state 5 [p_34_, cav].

Interestingly, the 1D projection of the MFEP of the Na^+^/Na^+^/Na^+^ system reveals a very high potential energy barrier between states 2 and 5 of ΔF^‡^ approx. 44 kJ/mol (Figure 3B and Figure S6). It is worth noting that this barrier for Na^+^ conduction is so high that it even exceeds the energy barrier generated by a Ba^2+^ inside the SF of a selective K^+^ channel.^60^ To quantify the difference in conductance between the K^+^/K^+^/K^+^ and Na^+^/Na^+^/Na^+^ systems, we used reaction rate theory to obtain relative transition rates for ion conductance: already in 1940 Kramer was able to obtain closed-form expressions to calculate the transition rate between wells in an over-damped system.^47^ By chaining jumps between wells along the MFEPs, we are able to attribute for each configuration with a relative transition rate. Following this approach, we calculated a dimensionless transition rate for all systems relative to the K^+^/K^+^/K^+^ system (Table 1). Unfortunately, for the Na^+^/Na^+^/Na^+^ system, we were not able to obtain a numerical solution for the ODE: here, the high energy barrier between states 2 and 5 led to rates exceeding the precision of 64 bit floating-point numbers. However, the highest barrier alone attributed for a rate that is several orders of magnitude lower than the overall rate of the K^+^/K^+^/K^+^ system (k(Na^+^/Na^+^/Na^+^)/k(K^+^/K^+^/K^+^) < 5.5 × 10^–10^,  Table 1 and Figure S10).

**Table 1.** Calculated transition rates for ion conduction in investigated systems. The transition rates are given relative to the transition rate of the K^+^/K^+^/K^+^ system.

System	Relative transition rates k
K^+^/K^+^/K^+^	1
Na^+^/Na^+^/Na^+^	5.5 10^–10^
K^+^/Na^+^/Na^+^	2 10^–4^
Na^+^/K^+^/K^+^	4.9 10^–6^
K^+^/K^+^	9.7 10^1^

We therefore expect that Na^+^ ions effectively block the pore of HCN channels for a substantial amount of time between conduction events in the absence of other ions. This hypothesis is in good agreement with experimental data showing only limited or no Na^+^ conductance in HCN channels in absence of K^+15^. The reason for this tendency of Na^+^ to block HCN channels is likely to be found in a stronger binding affinity of Na^+^ to carbonyl planes in the SF of these channels; this is made possible by the flexibility of the SF backbone,^8^ which allows the channel to “grapple” ions more strongly than a less flexible SF of a canonical K^+^ channel could do.

### Potassium Binding Facilitates Sodium Conductance

**Figure 4.**
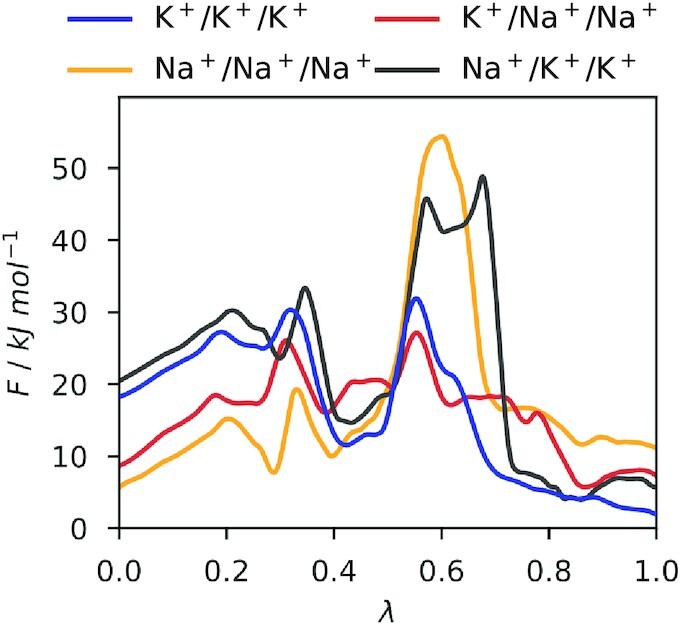
1D MFEPs of ion conduction in different systems: K^+^/K^+^/K^+^ (blue), Na^+^/Na^+^/Na^+^ (orange), K^+^/Na^+^/Na^+^ (red), Na^+^/K^+^/K^+^ (grey). Lambda is the reaction coordinate along the respective MFEPs.

Free energy calculations of Na^+^/Na^+^/Na^+^ predict an impaired conductance of HCN4 in pure Na^+^ solutions due to a large energy barrier between the two states [p_23_, s_4_] and [p_34_, cav]. In this condition Na^+^ acts like an effective channel-blocking agent and allows only a very limited conduction of Na^+^ ions. To test if the presence of K^+^ can recover, like in experiments,^61^ channel conductance in a Na^+^ containing solution, we calculated free energy profiles for mixed K^+^/Na^+^ ion conduction using the same method (Figure 4 and Figure S7–S9). Therefore, the two ions inside the SF and cavity (z_2_ and z_3_) were kept identical (either K^+^ or Na^+^), while the upper ion (z_1_) was replaced in each case with the other ion species (K^+^/Na^+^/Na^+^ and Na^+^/K^+^/K^+^ systems), respectively.

In both scenarios, free energy barriers between state 2 [p_23_, s_4_] and state 5 [p_34_, cav] are lowered by approximately −10 kJ/mol compared to the condition with a single ion species (compare K^+^/K^+^/K^+^ with K^+^/Na^+^/Na^+^ and Na^+^/Na^+^/Na^+^ with Na^+^/K^+^/K^+^, respectively). This in turn leads to transition rates that lie between the transition rates of the systems containing only K^+^ or only Na^+^ (Table 1). This is especially important for the Na^+^/K^+^/K^+^ system, where the height of the highest free energy barrier (ΔF^‡^ ∼ 34 kJ/mol) is now in a range compatible with the unitary conductance of 1 to 2 pS experimentally measured for native I_f_ channels of heterologous expressed HCN channels.^51^,^57–59^ The barrier is much lower than in the Na^+^/Na^+^/Na^+^ system, where free energy barriers were too high for a measurable conductance in experiments^15^ and MD simulations.^8^ Free energy profiles from calculations with mixed ions therefore suggest the following explanation for the experimentally observed K^+^-dependence of Na^+^ conduction: once a Na^+^ is bound to the SF, a newly arriving Na^+^ fails to initiate the conduction event that would favor the lower ion to leave the SF. This condition is equivalent to the aforementioned Na^+^/Na^+^/Na^+^ situation in which the energy barrier is too high for this step. However, when the newly arriving ion is a K^+^, the free energy barrier for the same event is lowered and the transition becomes more probable. In contrast, when the SF already contains a K^+^, a newly arriving ion can initiate a conduction event with a similar propensity independently on whether it is a K^+^ or Na^+^ ion. This enables the SF to support consecutive conduction from an initial state with a K^+^ bound but requires a K^+^ from the bulk solution once the SF has bound a Na^+^.

### Ion Permeation Strictly Follows Soft Know-On

**Figure 5.**
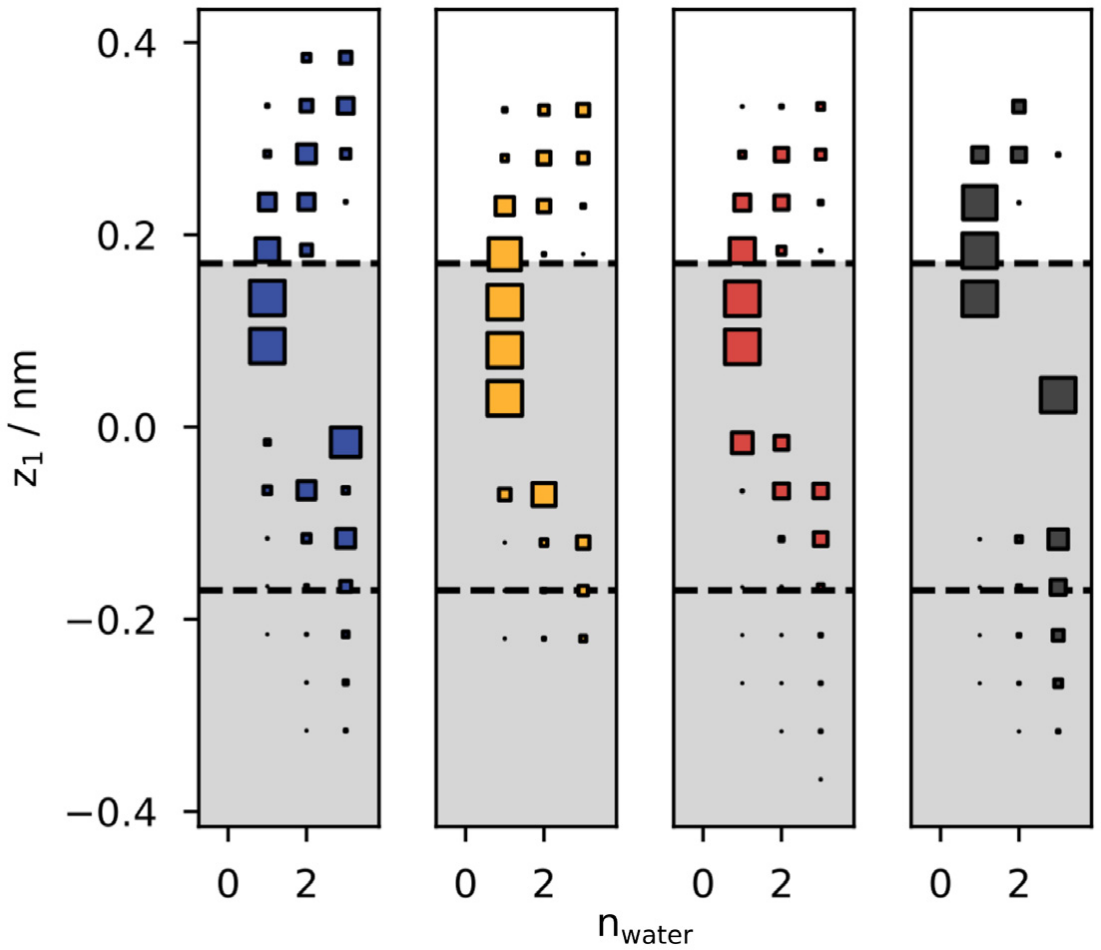
The number of water molecules between adjacent ions in the SF as function of the coordinate of the upper ion (z_1_). The volume of each square represents the density calculated from all frames along the given z coordinate. For clarity, the density is only shown for n = [0,3] because higher n correspond to unphysical configurations with ions far apart from each other (i.e., [ves, cav]). From left to right: K^+^/K^+^/K^+^ (blue), Na^+^/Na^+^/Na^+^ (orange), K^+^/Na^+^/Na^+^ (red), Na^+^/K^+^/K^+^ (grey). The shaded area corresponds to binding sites s_3_ and s_4_ and the upper and lower dashed lines correspond to carbonyl oxygen planes p_23_ and p_34_, respectively. It can be seen that there is always at least a single water molecule between adjacent ions (no density at n = 0).

For canonical K^+^ channels, two different mechanisms have been proposed for K^+^ ion conduction. In the “soft knock-on” mechanism, water and ions alternate inside the SF and one water molecule is conducted per ion conduction event.^18,62^ In contrast, the “direct knock-on” mechanism shows no water co-permeating with ions. Instead, ions can occupy adjacent binding sites directly 41, 54. An inspection of individual umbrella simulations revealed that even for simulations where we did not enforce ion-water alternation, (i.e., sampling starting from a state with a single ion inside of the SF, ions were always separated by at least one water molecule inside the SF and thus never occupied adjacent binding sites (Figure 5). Our simulations therefore suggest that ion permeation in HCN4 strictly follows a soft knock-on mechanism for all observed systems. These data are hence in good agreement with the general prediction that an exclusion of water is functionally coupled to high ion selectivity and high conductance in K^+^ channels^41^–features HCN do not have. Worth mentioning here is that simulations of HCN with co-conducting water also did not show the occasional escape of water molecules which were squeezing past ions. Also, configurations with more than one water at individual binding sites were not observed. These kinds of events have been reported from simulations of canonical SFs that enforced soft knock-on in umbrella simulations.^63^

## Conclusion

HCN channels are known for their slow, hyperpolarization-activated inward current. Important for their cellular function is that they conduct in physiological solution both K^+^ and Na^+^ with a ratio of about 4–6:1, but show only limited conductance in a pure Na^+^ solution.^2,3,15,16^ Structurally, the SF of HCN channels is different from other K^+^ channels in that it features only two classical carbonyl cages.^7,8^ In this study, we show that distinctly high energy barriers between SF binding sites are kinetically slowing down ion transitions through the filter and thus cause the low unitary channel conductance of HCN channels (about 1pS).^51,58^,^59^ Furthermore, our free energy calculations successfully predict a negligible unitary conductance of HCN4 in a pure Na^+^ solutions which arises from a very high free energy barrier between the state in which the filter switches from having two ions and one ion bound. This finding is in good agreement with experimental recordings showing that native I_h_ currents and HCN channels loos their ability to carry an inward Na^+^ current in an external buffer with only sodium.^15,61^,^64–66^ This effective “block” of the channel by a single bound Na^+^ ion can be more easily released by an incoming K^+^ than another Na^+^. This explains experimental data showing that the ability of native If channels and HCN channels to conduct Na^+^ is augmented by the presence of K^+^ ion in the external buffer.^15,61^ In contrast, when K^+^ is bound in the SF, both Na^+^ and K^+^ ions can equally well initiate the next conduction event. All this favors the experimentally well established preference of K^+^ over Na^+^ conduction in these channels and reduces conductance of Na^+^ in the absence of potassium.^15^ Furthermore, our simulations strictly follow a soft know-on conduction mechanism in which ions are separated by exactly by one water molecule inside the SF. This mechanism has been proposed in the context of other channels as a condition that also favors low conduction rates and low cation selectivity in general.^41,67^

## Supplementary Material

zqac019_Supplemental_Figures_and_Table

## Data Availability

The data underlying this article are available at Zenodo, https://doi.org/10.5281/zenodo.6407763.

## Supplementary Material

Additional figures and calculated free energy differences are provided as supporting material.
