# Supplementary material for: Weak Cation Selectivity in HCN Channels Results From K+-Mediated Release of Na+ From Selectivity Filter Binding Sites
Source: Function (Oxf). 2022 Apr 22;3(3):zqac019. doi: 10.1093/function/zqac019 (PMC9492253; doi:10.1093/function/zqac019)
Supplement: zqac019_Supplemental_Figures_and_Table [file zqac019_supplemental_figures_and_table.zip › Supplement Figure 1.docx]

**Supplement Figure 1**


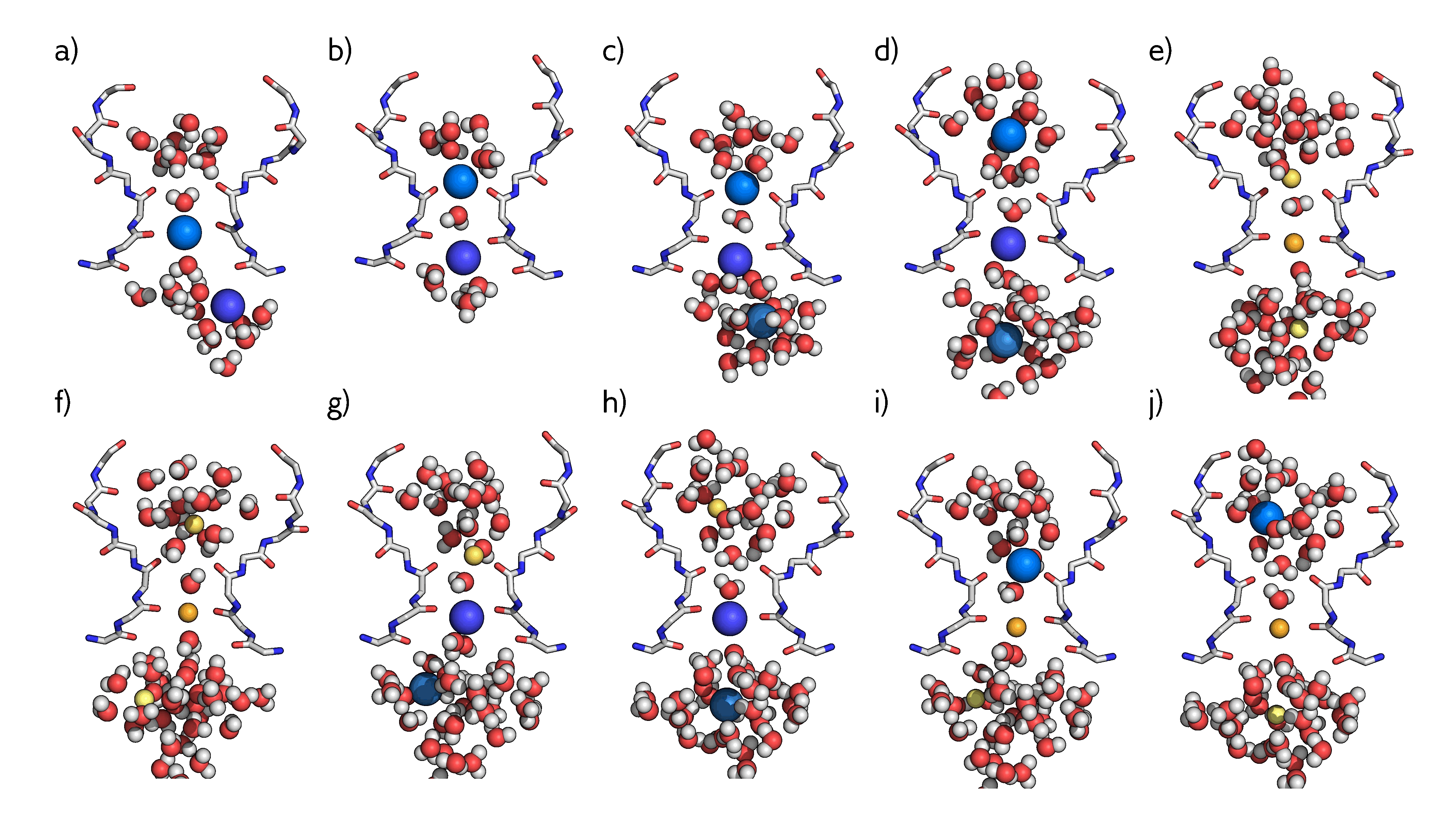


**Fig. S1.** SF snapshots of configurations used as starting points for self-learning adaptive umbrella simulations: a-b) K^+^/K^+^, c-d) K^+^/K^+^/K^+^, e-f) Na^+^/Na^+^/Na^+^, g-h) Na^+^/K^+^/K^+^, i-j) K^+^/Na^+^/Na^+^. The remaining protein is not shown for clarity. Potassium and sodium ions are shown as blue and orange spheres, respectively. Water molecules in close proximity to ions are shown as red/white spheres and the SF is shown as colored sticks.
