# Supplementary material for: Weak Cation Selectivity in HCN Channels Results From K+-Mediated Release of Na+ From Selectivity Filter Binding Sites
Source: Function (Oxf). 2022 Apr 22;3(3):zqac019. doi: 10.1093/function/zqac019 (PMC9492253; doi:10.1093/function/zqac019)
Supplement: zqac019_Supplemental_Figures_and_Table [file zqac019_supplemental_figures_and_table.zip › Supplement Figure 10.docx]

**Supplement Figure 10**


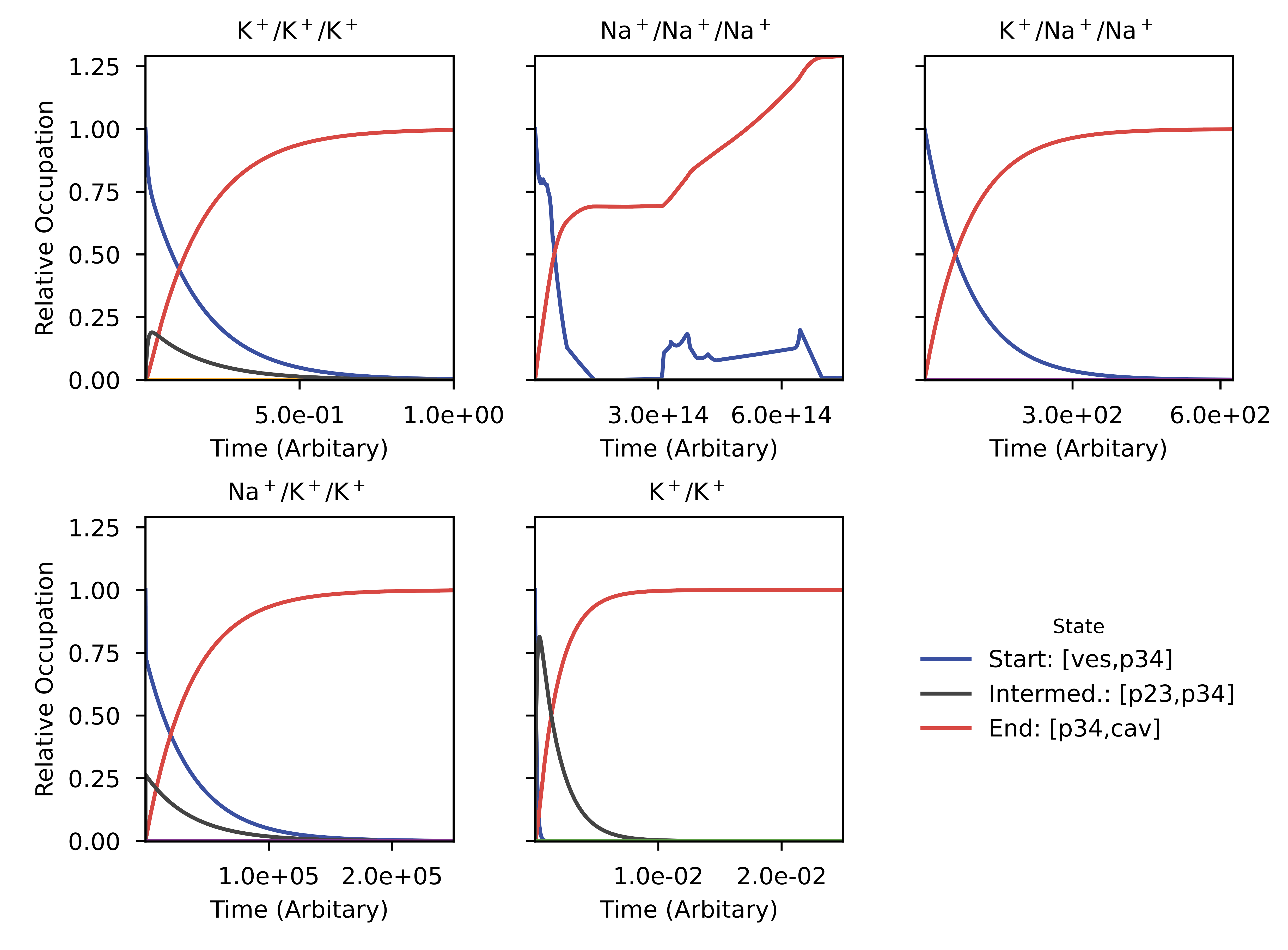


**Fig. S10.** Relative occupation numbers of relevant states of the MFEPs over simulation time of the ODEs. It can be seen that all systems expect the Na^+^/Na^+^/Na^+^ system transition to a relative occupation of 1.00 for the end state [p_23,cav_]. In contrast, the Na^+^/Na^+^/Na^+^ system does not reach this state over a long simulation time (1 × 10^15^). Instead, limited floating point precision leads to a relative occupation > 1.00 because of the high energy barrier between the first and last state.
