# Supplementary material for: Weak Cation Selectivity in HCN Channels Results From K+-Mediated Release of Na+ From Selectivity Filter Binding Sites
Source: Function (Oxf). 2022 Apr 22;3(3):zqac019. doi: 10.1093/function/zqac019 (PMC9492253; doi:10.1093/function/zqac019)
Supplement: zqac019_Supplemental_Figures_and_Table [file zqac019_supplemental_figures_and_table.zip › Supplement Figure 2.docx]

**Supplement Figure 2**


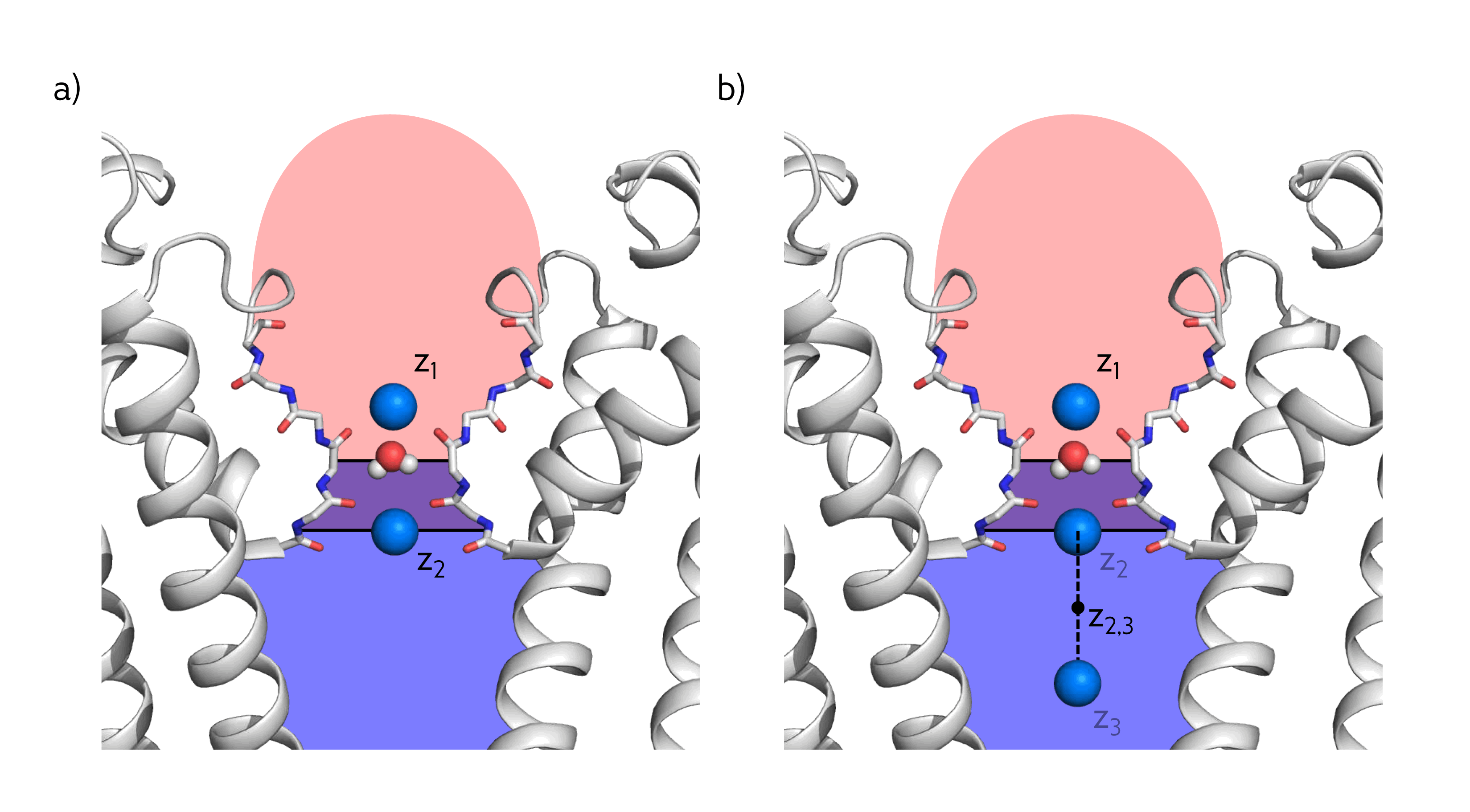


**Fig. S2.** Depiction of the umbrella sampling scheme. a) 2 ions system: z_1_ and z_2_ describe the z-coordinate of the upper and lower ion relative to the s_3_ site, respectively. z_1_ was limited to sample the region shaded in red and z_2_ sampled the region shaded in blue. b) 3 ions system: z_2,3_ represents the centroid between z_2_ and z_3_.
