# Supplementary material for: Weak Cation Selectivity in HCN Channels Results From K+-Mediated Release of Na+ From Selectivity Filter Binding Sites
Source: Function (Oxf). 2022 Apr 22;3(3):zqac019. doi: 10.1093/function/zqac019 (PMC9492253; doi:10.1093/function/zqac019)
Supplement: zqac019_Supplemental_Figures_and_Table [file zqac019_supplemental_figures_and_table.zip › Supplement Figure 3.docx]

**Supplement Figure 3**


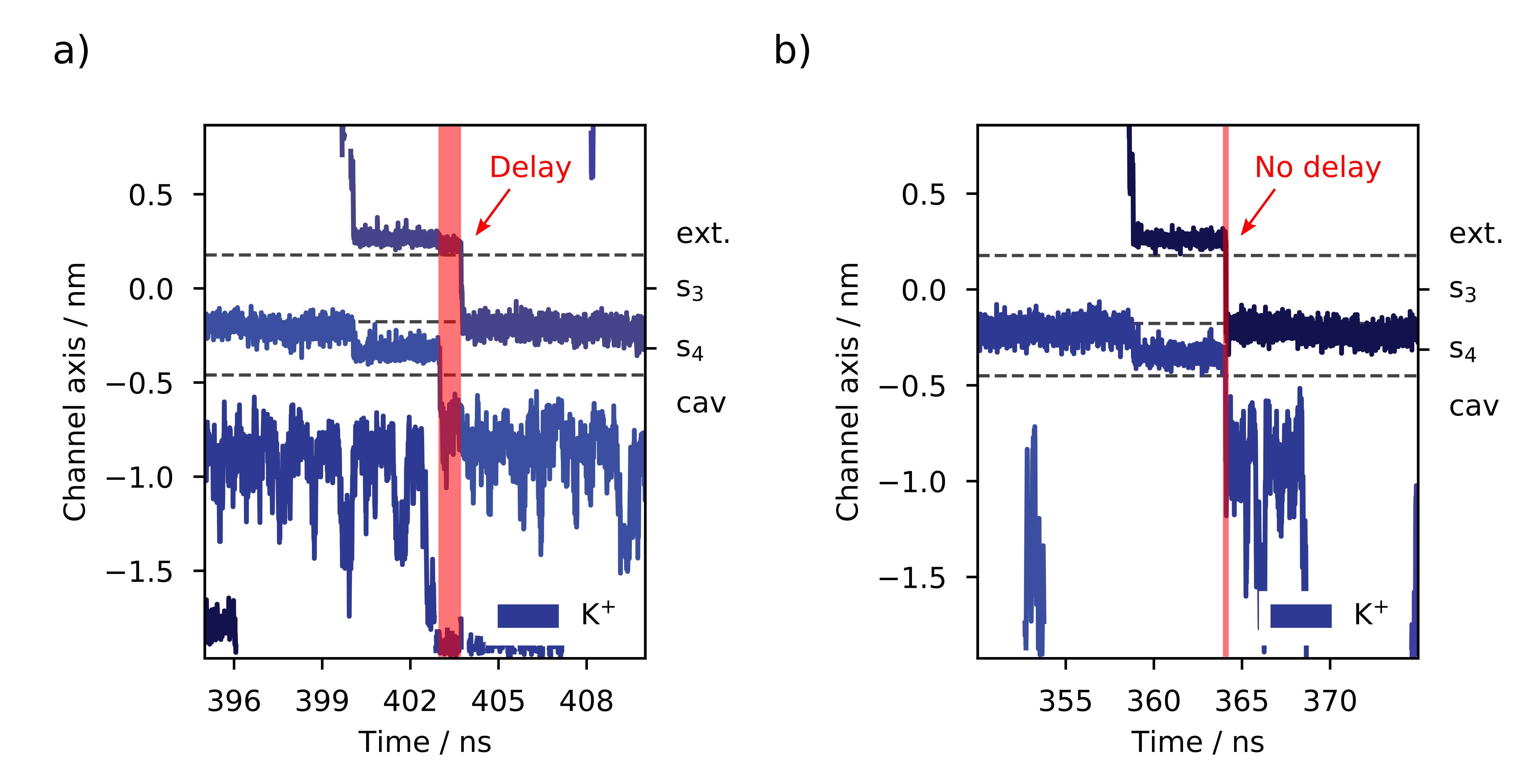


**Fig. S3.** Ion trajectories for K^+^ conduction under hyperpolarizing voltages. Different shades of blue correspond to different K^+^ ions and gray dashed lines represent carbonyl oxygens of the SF. a) Movement of the upper ion is delayed by approx. 1 ns (red area) after the lower ion left the SF. b) Ions move in concert with no observable delay. Simulation parameters have been reported previously (Saponaro et al. 2021).
