# Supplementary material for: Weak Cation Selectivity in HCN Channels Results From K+-Mediated Release of Na+ From Selectivity Filter Binding Sites
Source: Function (Oxf). 2022 Apr 22;3(3):zqac019. doi: 10.1093/function/zqac019 (PMC9492253; doi:10.1093/function/zqac019)
Supplement: zqac019_Supplemental_Figures_and_Table [file zqac019_supplemental_figures_and_table.zip › Supplement Figure 4.docx]

**Supplement Figure 4**


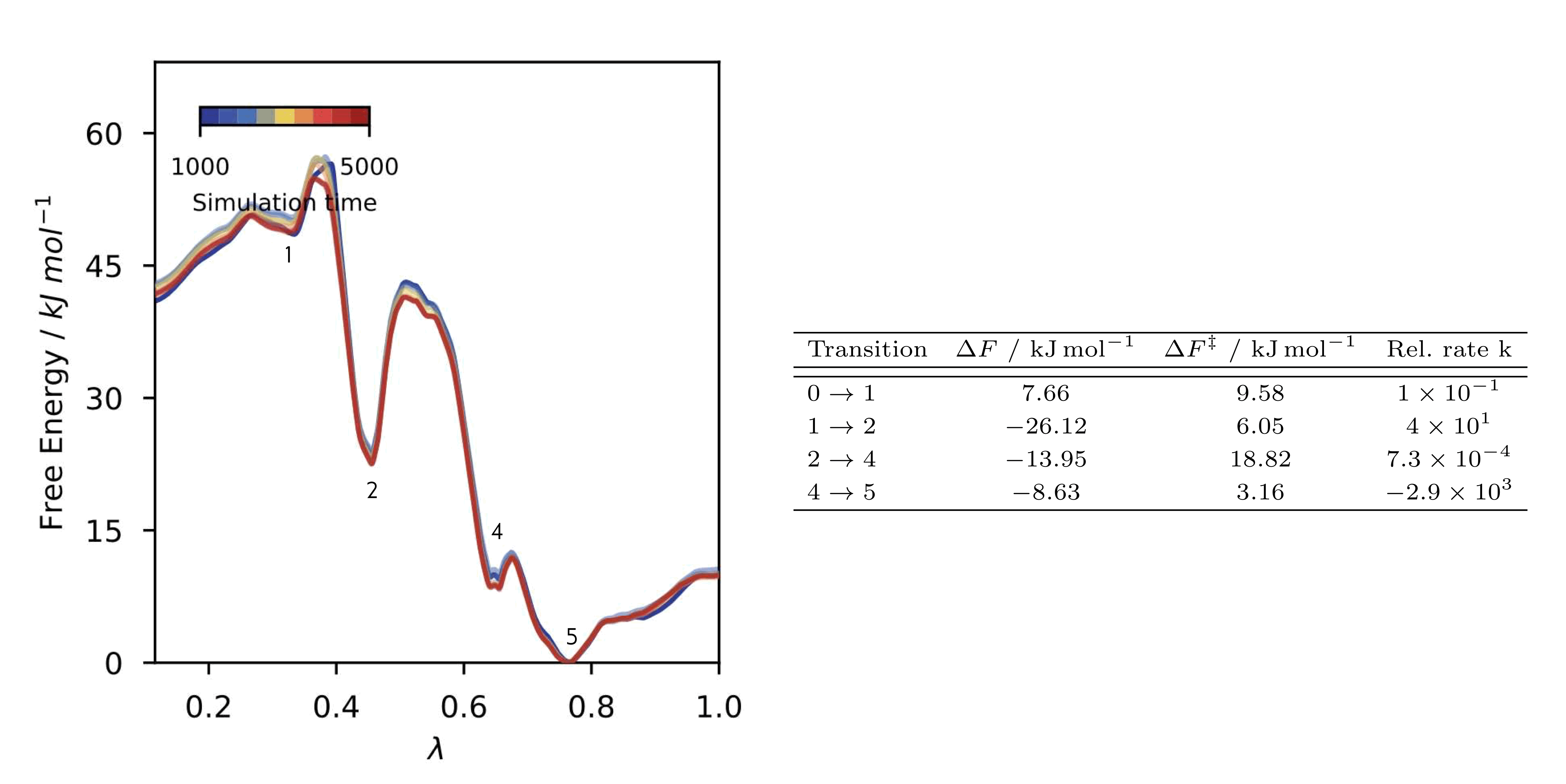


**Fig. S4.** Free energy of the K^+^/K^+^ system. a) Convergence of the MFEP through the 2D PMF. Colors represent the free energy path through the PMF in 500 ps intervals starting with the interval from 500 ps to 1000 ps. For each plot, the free energy path was extracted from the full simulation and was then superimposed on PMFs calculated with reduced simulation time. b) Calculated free energy differences and Kramer transition rates between minima Fi → j and the height of the highest energy barrier for corresponding transitions F^‡^i → j.
