# Supplementary material for: Weak Cation Selectivity in HCN Channels Results From K+-Mediated Release of Na+ From Selectivity Filter Binding Sites
Source: Function (Oxf). 2022 Apr 22;3(3):zqac019. doi: 10.1093/function/zqac019 (PMC9492253; doi:10.1093/function/zqac019)
Supplement: zqac019_Supplemental_Figures_and_Table [file zqac019_supplemental_figures_and_table.zip › Supplement Figure 7.docx]

**Supplement Figure 7**


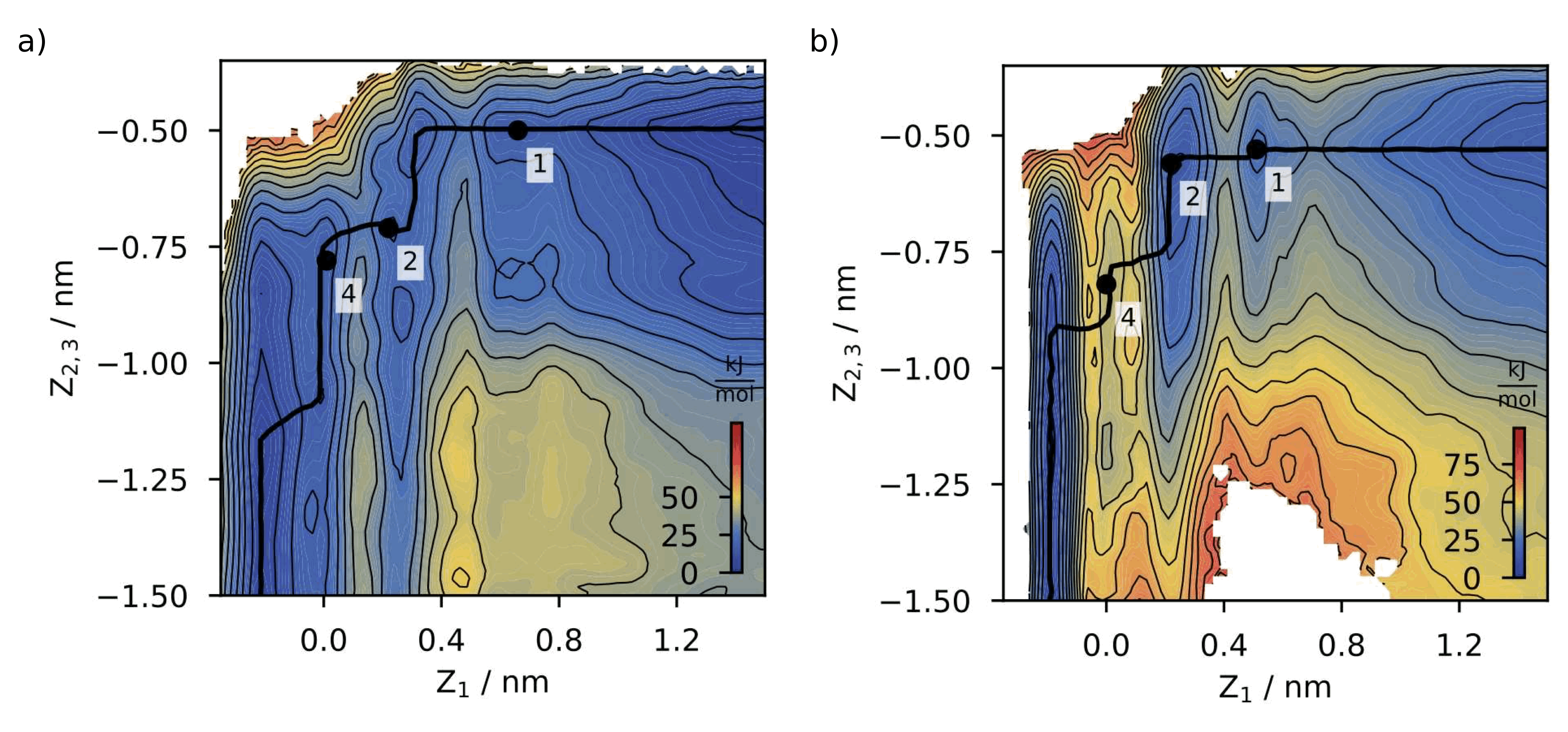


**Fig. S7.** Free energy landscape of ion conduction with 3 ions in the a) K^+^/Na^+^/Na^+^ and b) Na^+^/K^+^/K^+^ system. The Free energy is shown as function of the position of three ions z_1_ and z_2,3_ along the principal axis of the channel. The s_3_ binding site is located at Z=0 nm and more positive values of z correspond to locations at the extracellular side. The black solid line represents the MFEP through the 2D landscape. Local minima of the MFEP are labeled numerically (1-5). Contours represent a free energy difference of 5kJmol^-1^.
