# Supplementary figures and images for: Weak Cation Selectivity in HCN Channels Results From K+-Mediated Release of Na+ From Selectivity Filter Binding Sites
Source: Function (Oxf). 2022 Apr 22;3(3):zqac019. doi: 10.1093/function/zqac019 (PMC9492253; doi:10.1093/function/zqac019)

**Table S1.** Overview over umbrella sampling simulation parameters and starting configurations.


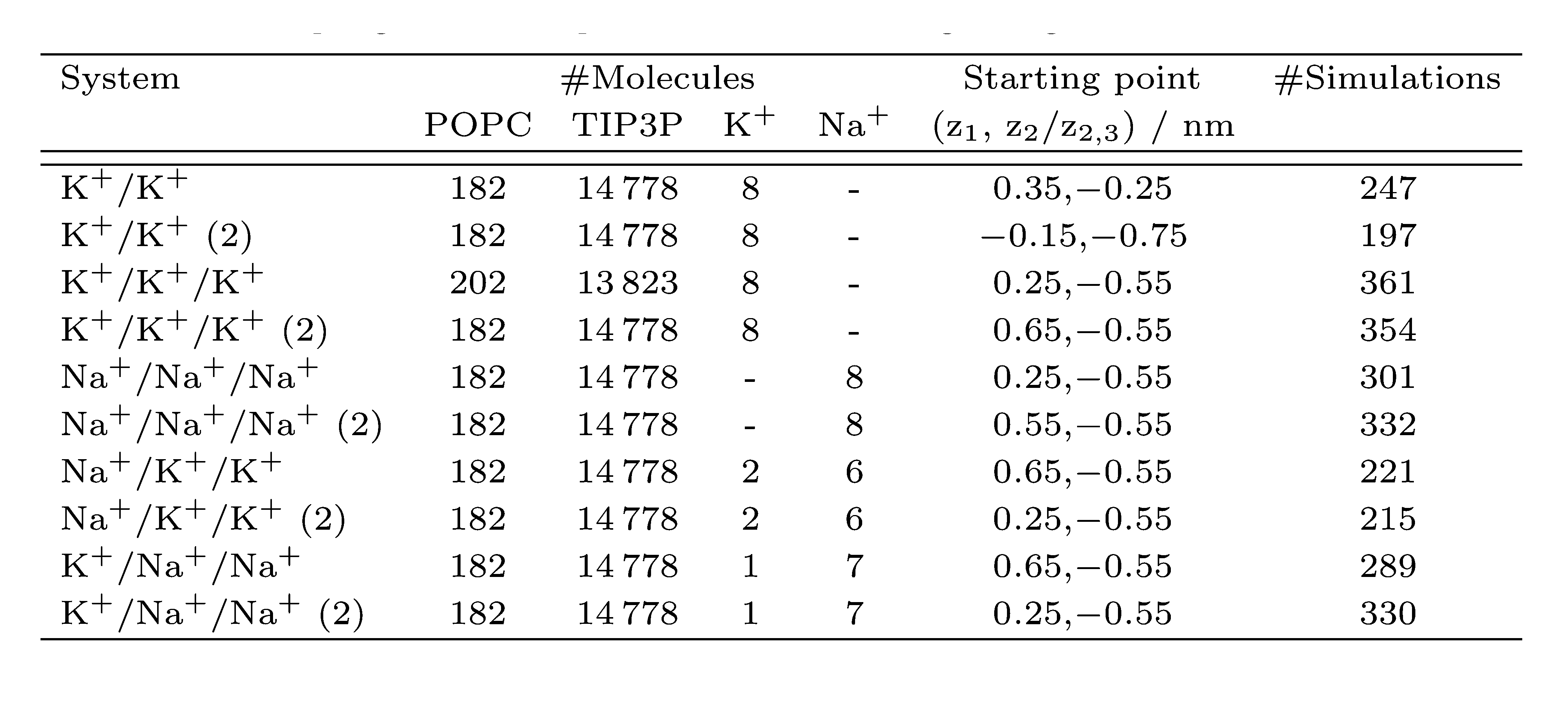

Supplement: zqac019_Supplemental_Figures_and_Table [file zqac019_supplemental_figures_and_table.zip › Suplement table.docx]
